# A Robust Rotation-Equivariant Feature Extraction Framework for Ground Texture-Based Visual Localization

**DOI:** 10.3390/s25123585

**Published:** 2025-06-06

**Authors:** Yuezhen Cai, Linyuan Xia, Ting On Chan, Junxia Li, Qianxia Li

**Affiliations:** School of Geography and Planning, Sun Yat-sen University, Guangzhou 510275, China; caiyzh5@mail2.sysu.edu.cn (Y.C.); xialiny@mail.sysu.edu.cn (L.X.); chantingon@mail.sysu.edu.cn (T.O.C.); lijx373@mail2.sysu.edu.cn (J.L.)

**Keywords:** visual localization, ground texture, keypoint detection, feature extraction, deep learning

## Abstract

Ground texture-based localization leverages environment-invariant, planar-constrained features to enhance pose estimation robustness, thus offering inherent advantages for seamless localization. However, traditional feature extraction methods struggle with reliable performance under large-scale rotations and texture sparsity in the case of ground texture-based localization. This study addresses these challenges through a learning-based feature extraction framework—Ground Texture Rotation-Equivariant Keypoints and Descriptors (GT-REKD). The GT-REKD framework employs group-equivariant convolutions over the cyclic rotation group, augmented with directional attention and orientation-encoding heads, to produce dense keypoints and descriptors that are exactly invariant to 0–360° in-plane rotations. The experimental results for ground texture localization show that GT-REKD achieves 96.14% matching in pure rotation tests, 94.08% in incremental localization, and relocalization errors of 5.55° and 4.41 px (≈0.1 cm), consistently outperforming baseline methods under extreme rotations and sparse textures, highlighting its applicability to visual localization and simultaneous localization and mapping (SLAM) tasks.

## 1. Introduction

With the rapid growth of the Internet of Things (IoT) and artificial intelligence, accurate and seamless indoor–outdoor localization has become an essential infrastructure for the digital economy and society [1,2,3,4]. Seamless indoor–outdoor localization technologies must achieve accurate, real-time, and smooth transitions between open outdoor environments and complex indoor settings to fulfill the rigorous requirements of centimeter-level accuracy and low latency in applications such as industry applications [5], robotic navigation [6], and autonomous driving [7,8]. Currently, mainstream localization approaches, as well as their design characteristics and performance boundaries, include the following:

- Satellite navigation (GNSS/SBAS): Offers global coverage but is significantly limited in urban canyons and indoor areas due to signal blockage and interference [9,10,11].
- Active localization (UWB, Radar, RFID, etc.): Employs nanosecond-level wireless pulses or radio frequency signals to achieve centimeter-level accuracy suitable for complex environments; however, these methods entail high hardware costs, elevated energy consumption, and susceptibility to multipath effects and obstacle interference [12,13,14,15].
- Passive localization (Wi-Fi, Bluetooth, cellular signals, geomagnetic fields, etc.): Characterized by low deployment costs and broad coverage, these methods depend heavily on dense signal infrastructure, typically resulting in meter-level positioning accuracy [16,17,18,19,20,21].
- Inertial/Visual/LiDAR-based localization: Utilizes inertial measurement units (IMUs), visual sensors, or LiDAR for environment perception without pre-installed infrastructure; nevertheless, these methods face challenges such as sensitivity to lighting conditions, sparse textures, cumulative drift errors, and issues in map storage and cross-environment adaptation [22,23,24,25,26].

**Figure 1:**
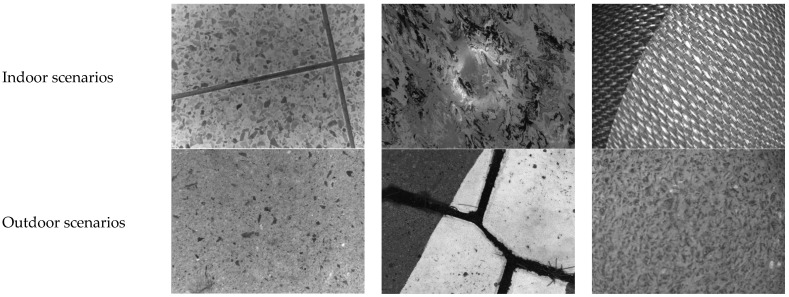
Examples of ground texture images for indoor–outdoor visual localization.

Focusing on the limitations inherent in existing indoor–outdoor localization technologies, our study specifically emphasizes low-cost and high-precision positioning methods for ground robots utilizing general-purpose cameras based on ground texture [27]. Ground textures—characterized by minute cracks, imperfections, and material variations—offer dense, distinctive features ideal for precise visual localization. As shown in Figure 1, the ground texture localization method uses only a single downward-facing camera with autonomous illumination, eliminating reliance on external infrastructure, minimizing interference from environmental dynamics, signal loss, and varying lighting conditions, and inherently protecting privacy by avoiding sensitive imagery. These advantages enable practical, seamless transitions between indoor and outdoor environments, making the framework ideal for applications in factories, warehouses, parking facilities, autonomous vehicles, and low-altitude, ground-facing mobile robots (e.g., robotic vacuum cleaners).

Ground texture-based localization methods can be broadly classified into two categories: absolute localization and incremental localization. Absolute localization involves an offline phase for map creation using prior pose information and an online phase for matching live images with the map to estimate the current pose. Kozak and Alban [28] introduced a framework for absolute localization using offline road surface image collection and online feature point matching. Their method demonstrated deplorability and practicality at high speeds (up to 130 km/h) with centimeter-level accuracy across various road materials, though it required GNSS constraints and operated at only 10 Hz. Subsequent improvements by Chen et al. [29] incorporated SURF features, optical flow, and SLAM modules to enhance map construction and localization without prior constraints, while integrating IMUs and the Extended Kalman Filter (EKF) to improve accuracy.

An advancement in absolute localization was introduced by Zhang et al. [30] with the “Micro-GPS” module, which balances accuracy and speed by combining high-response SIFT sub-features, PCA-based dimensionality reduction, and kernel density analysis for rapid and precise localization. Building on this, Wilhelm and Napp [31] proposed a lightweight approach that addressed limitations in PCA for binary features (e.g., ORB) and accelerated matching with KD-tree structures. Further developments by Wang et al. [32] and Sheng et al. [33] explored mechanisms to improve real-world deployment, including binary descriptor-based bag-of-words models, online map updates, and dual-stage matching strategies for enhanced accuracy and reduced error rates in large-scale environments.

Incremental localization, on the other hand, emphasizes SLAM in unknown environments. Hart et al. [34] pioneered SLAM using ground textures within the ORB-SLAM framework, dividing localization into local visual odometry and loop closure optimization. However, the method required offline map collection for training and relied on manually tuned thresholds for loop closure filtering, limiting its generalizability. Xu et al. [35] introduced a remote sensing-inspired approach using Fourier transforms to compute frequency-domain representations and improve frame-to-frame matching in weak-texture regions. While effective in addressing certain challenges, their method struggled with loop closure detection due to assumptions about image overlap.

Most existing approaches rely on feature point detection for localization. However, in ground texture localization tasks, classical detectors—such as Harris corner, FAST, SIFT, and ORB—suffer from feature omission, mismatches, and non-uniform spatial distribution when the camera undergoes significant viewpoint changes. Schmid et al. [27,36] present the first extensive evaluation of feature extraction methods for ground texture localization and found that most handcrafted keypoint detection methods performed not well, and the quality of descriptors determines the accuracy of keypoint matching. By contrast, deep learning-based methods leverage end-to-end Convolutional Neural Networks to learn high-dimensional, discriminative feature representations, exhibiting superior robustness to noise and illumination variations and thus maintaining high matching accuracy in texture-sparse or low-contrast scenes. Nevertheless, these approaches demonstrate limited adaptability to large-angle rotations and scale changes. In ground texture localization, the camera frequently experiences severe yaw rotations, resulting in substantial image rotation distortions that further exacerbate the challenges of feature extraction and matching.

To address large-angle rotations and sparse textures in ground texture localization, we propose Ground Texture Rotation-Equivariant Keypoints and Descriptors (GT-REKD), the first rotation-equivariant framework tailored to ground texture localization. GT-REKD embeds rotation-equivariant convolutional kernels [37] into its network backbone and augments them with an orientational attention mechanism, thereby ensuring inherent invariance to arbitrary rotations while enhancing discrimination of fine texture details. The remainder of this paper first describes the overall architecture and key modules of GT-REKD and then presents quantitative evaluations and ablation studies on multiple ground texture datasets to demonstrate its improved matching robustness and localization accuracy in scenarios characterized by large rotations, repeated patterns, and sparse textures. Its core contributions can be concluded as follows:

- Rotation-equivariant convolutional backbone: GT-REKD embeds cyclic-group convolutions into the feature extractor, yielding intrinsic invariance to arbitrary in-plane rotations across multiple spatial scales while preserving computational efficiency.
- End-to-end learnable orientation head: GT-REKD decouples orientation encoding from descriptor learning, ensuring consistent keypoint direction labels and robust matching.
- Extensive multi-scenario evaluation with leading performance: Extensive multi-scenario evaluation, including pure rotation matching, incremental localization, and absolute relocalization across diverse ground materials (brick, asphalt, concrete, and gravel), demonstrates GT-REKD’s leading performance, achieving 96.14% matching accuracy in pure rotation, 94.08% in incremental localization, and average relocalization errors of 5.55°/4.41 px, consistently outperforming baseline methods, such as ORB, SIFT, SuperPoint, etc.

## 2. Preliminaries

### 2.1. Pose Estimation on Ground Texture

Ground texture localization benefits from the fact that all visual features lie on a common planar surface. This constraint reduces the six-degree-of-freedom pose estimation problem to a four-degree one and permits a homography-based formulation. Given a set of matched feature pairs $\{(x_{i},x_{i}^{\prime }){\}}_{i=1}^{N}$ in homogeneous image coordinates, the relative motion between two frames is modeled by a planar projective transformation ${x_{i}}^{\prime }≃Hx_{i}$.

The homography H is recovered by minimizing the reprojection error of at least four point correspondences. We adopt the normalized Direct Linear Transform (DLT) to form a linear system Ah=0, solve it via singular-value decomposition, and enforce det(H)>0. To suppress outliers arising from mismatches or non-planarity, RANSAC iteratively re-estimates H and selects the hypothesis with the lowest consensus error. This procedure yields a robust frame-to-frame planar motion estimate.

Let $H_{j|i}$ denote the homography from image *i* to *j*. Chaining successive transforms yields each frame’s planar pose

(1) $$H_{j}=H_{j|i}H_{i}.$$

Because H is expressed in pixel units, a metric upgrade is required. Decomposing it with the intrinsic matrix K gives the following:

(2) $$K^{-1}H=\left[\begin{array}{ccc} r_{1} & r_{2} & t \end{array}\right],$$

where $r_{1}$, $r_{2}$ are orthonormal rotation columns and t is the translation in pixel scale. The third rotation axis and full rotation matrix are as follows:

(3) $$r_{3}=r_{1}\times r_{2},\;\;R_{w}=\left[\begin{array}{ccc} r_{1} & r_{2} & r_{3} \end{array}\right].$$

Absolute scale is resolved from the known camera height *h* above the ground plane:

(4) $$\lambda =\frac{h}{t_{z}},\;\;t_{w}=\lambda t.$$

Finally, the metric SE(3) pose of each frame is as follows:

(5) $$T_{w}=\left[\begin{array}{cc} R_{w} & t_{w}\\ 0^{T} & 1 \end{array}\right].$$

This formulation supplies globally consistent metric poses while avoiding full 3D reconstruction, enabling efficient incremental localization and global alignment on planar ground texture sequences.

The planar homography formulation eliminates the need for 3D reconstruction while preserving metric consistency, enabling efficient incremental localization and global alignment. Empirically, this approach achieves sub-pixel reprojection error and centimeter-level absolute accuracy on benchmark ground texture datasets, providing a lightweight yet reliable backbone for seamless indoor–outdoor navigation pipelines.

### 2.2. Group-Equivariant Convolutions

At its core, a group-equivariant convolution (G-Conv) is a type of convolutional layer designed to incorporate known symmetries into a neural network. The “group” in its name refers to a mathematical group of transformations (e.g., rotations, translations, reflections). Equivariance means that if the input to the layer is transformed by an element of this group, the output of the layer is also transformed in a corresponding way.

Imagine having an image of a cat: if we rotate the image, a standard Convolutional Neural Network (CNN) might struggle to recognize it as the same cat because the learned features are not inherently rotation-aware. G-Conv aims to solve this. If you rotate the input image, the feature maps produced by a G-Conv layer will be rotated versions of the feature maps produced for the original image. This makes the network inherently robust to these specific transformations.

For an image $f:R^{2}\to R^{C_{\mathrm{in}}}$ and kernel $\psi :R^{2}\to R^{C_{\mathrm{in}}\times C_{\mathrm{out}}}$, the conventional convolution is as follows:

(6) $$(f*\psi )(x)=\sum_{y\in Z^{2}}\;f(y)\psi (x-y),$$

which satisfies translational equivariance.

(7) $$L_{t}[f*\psi ]=(L_{t}f)*\psi \;with\;L_{t}f(x)=f(x-t).$$

On the other hand, let *G* be a discrete symmetry group acting on $R^{2}$, and denote by $f:G\to R^{C_{in}}$ a lifted feature map. The G-Conv is as follows:

(8) $$(f{*}_{-}G\psi )(g)=\sum_{h\in G}\;f(h)\psi \left(g^{-1}h\right),g\in G,$$

with kernel $\psi :G\to R^{C_{in}\times C_{out}}$.

Because convolution is now performed over the group itself, we obtain full G-equivariance:

(9) $$L_{g}\left[f{*}_{G}\psi \right]=\left(L_{g}f\right){*}_{G}\psi ,\forall g\in G,$$

where $\left(L_{g}f)(h)=f(g^{-1}h\right)$. Therefore, G-Conv addresses this limitation by constructing feature maps and kernels over group *G*, enabling the network to be equivariant to a wider range of transformations. This leads to improved generalization and efficiency, especially in tasks where such symmetries are prevalent.

## 3. Methodology

The proposed network integrates modern characteristics from unsupervised keypoint detection networks such as R2D2 [38] and D2-Net [39], including simplicity, end-to-end output, and avoiding explicit design of keypoint distributions. To further enhance rotational robustness, GT-REKD incorporates G-Conv, enabling intrinsic equivariance to rotations in the feature extraction stage. It also draws inspiration from network architectures like SuperPoint [40] and SiLK [41], which simultaneously detect keypoints, descriptors, and design principles from rotationally invariant feature networks such as RELF [42] and RIDE [43]. GT-REKD aims to unify simplicity and flexibility, leveraging traditional local feature methods and modern self-supervised learning techniques to efficiently detect and describe image keypoints without requiring labeled data.

### 3.1. Network Architecture

**Figure 2:**
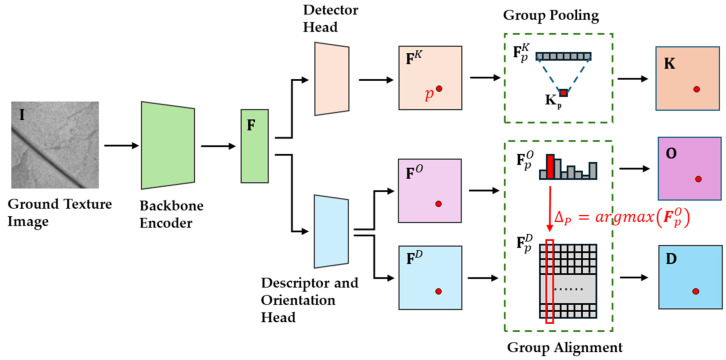
GT-REKD network architecture produces dense keypoints (K), orientation histograms (O), and rotation-equivariant descriptors (D).

As illustrated in Figure 2, GT-REKD is structured to ensure both rotation-equivariance and descriptor discriminability through four core components:

(a) Group-equivariant encoder backbone

The ground texture image I is encoded by a stack of cyclic-group convolution layers, producing a feature tensor map F that is intrinsically equivariant to any in-plane rotation in the |G|-element group.

(b) Detector head—($F^{K}\to K$)

A lightweight MLP converts F into a group score tensor $F^{K}$. Orientation-channel max-pooling (group pooling) yields the scalar keypoint confidence map K with the element, facilitating reliable detection even under large rotations and sparse textures.

(c) Orientation head—($F^{O}\to O$)

A parallel branch outputs tensor $F^{O}$**,** which represents a dense orientational histogram. Channel-wise softmax normalizes each location *p* into an |G|-bin orientation histogram $F_{p}^{O}$. The dominant bin $\Delta_{p}$ gives the canonical orientation used for descriptor alignment, and the orientation map O stores the orientation of every *p* after softmax normalization.

(d) Descriptor head—($F^{D}\to D$)

A third branch computes the raw descriptor tensor $F^{D}$. A cyclic shift by $\Delta_{p}$ realigns the descriptor channels, producing the final L2-normalised descriptor map D.

These four components jointly ensure that GT-REKD (i) detects keypoints and assigns confidence in a rotation-equivariant way, (ii) estimates per-pixel canonical orientations, and (iii) outputs orientation-aligned, highly discriminative descriptors suitable for robust matching and localization even under extreme rotations and sparse textures. Detailed algorithmic formulations are provided in Section 3.2, Section 3.3 and Section 3.4.

### 3.2. Feature Extraction Based on Rotational Equivariance

**Figure 3:**
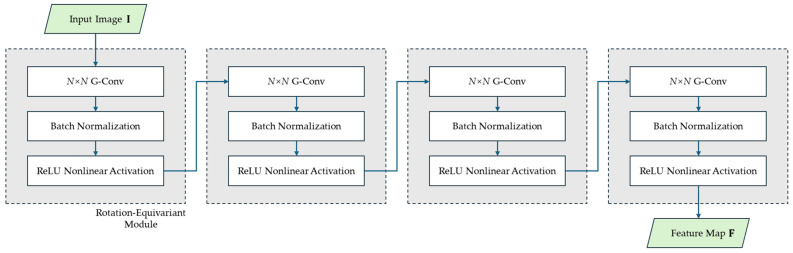
Implementation details of the backbone encoder in GT-REKD.

As the feature extractor, this paper adopts a backbone encoder network (similar to the VGG structure [44]) to learn high-dimensional features from image $I\in R^{H\times W}$. Figure 3 illustrates the details of the backbone encoder, which processes the input image through a cascade of four rotation-equivariant modules, each module comprising an *N*×*N* G-Conv layer, batch normalization layer, and ReLU activation layer to produce the final feature map. It is worth noting that the network does not employ max-pooling or padding operations as most convolution operations go: this design preserves feature density while preventing padding from introducing learnable corner and edge features that could lead to model overfitting. However, this approach reduces the image dimensions: for a 5 × 5 G-Conv kernel, each convolution operation reduces both the width and height of the image by 4 pixels. Additionally, the backbone network can be easily replaced, allowing for experimentation with a variety of network configurations.

The G-Conv kernels operate on a cyclic rotation group |G|, where the value of |G| represents the order of the rotation group. For example, a rotation group with |G| = 8 indicates that the network learns image features across eight equivariant rotational orientations. While these convolutional kernels introduce additional training overhead, they maintain the same inference speed as standard convolutional kernels during the testing phase, ensuring the simplicity and efficiency of the network architecture.

After the dense feature map $F\in R^{\left(C\times \left|G\right|\right)\times H^{\prime }\times W^{\prime }}$ are extracted through the backbone network, the same feature receptive field is fed into both the keypoint and descriptor detection heads. For the keypoints, *N*×*N* follows a 1×1 rotation-equivariant module (see Figure 3) and reduces the number of channels in the group transformation set to a single dimension, resulting in a keypoint score map $F^{K}\in R^{\left|G\right|\times H\prime \times W\prime }$. For descriptors, the detection head consists of two components after another rotation-equivariant module: (i) a 1 × 1 convolutional module continues learning features to generate a dense descriptor map $F^{D}\in R^{\left(C\times \left|G\right|\right)\times H\prime \times W\prime }$; (ii) an attention mechanism is introduced to learn the orientational properties of descriptors, forming a dense orientational histogram map $F^{O}\in R^{\left|G\right|\times H\prime \times W\prime }$. This enables the estimation of keypoint orientation and further processing of the descriptors with group alignment operation, which we will discuss in the next section.

### 3.3. Descriptor and Keypoint Extraction Based on Rotational Equivariance

**Figure 4:**
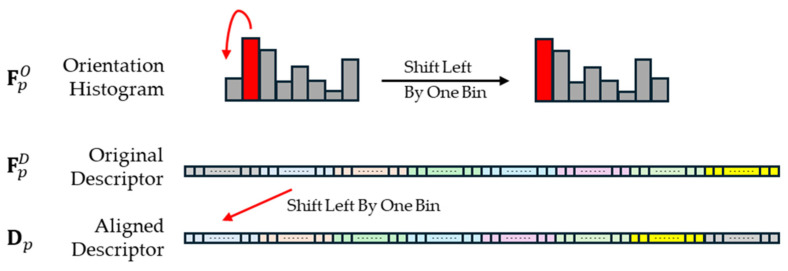
Detailed explanation of group alignment operation in Figure 2 (|G| = 8): the highest bin of the orientation (marked as red) histogram indicates the principal orientation; therefore, the descriptor elements associated with this orientation are circularly shifted to the front, ensuring that all descriptors in each group share a common principal orientation. The colored segments in the original descriptor represent different oriented group partitions of raw descriptors, and the red arrow indicates the circular left-shift operation used to align descriptors.

For a point *p* in the dense feature map, its corresponding orientational histogram $F_{p}^{O}\in R^{\left|G\right|}$ can be obtained from $F^{O}$. This histogram represents the response values to different rotational transformations g in the rotation group. After applying softmax normalization to $F_{p}^{O}$, the dimension with the highest response value, $\Delta_{p}$, is selected as the estimated orientation of the point. Simultaneously, the descriptor at point *p* can be represented as $F_{p}^{D}\in R^{\left(C\times \left|G\right|\right)}$, which can be considered as a one-dimensional tensor of length C×|G|. Based on the size of the rotation group, $\left|G\right|$, the descriptor is unfolded and concatenated in a two-dimensional plane, where $F_{p}^{D}$ aligns with the dimensions of the group transformations and corresponds one-to-one with the orientational histogram $F_{p}^{O}$. The group transformation dimension corresponds to the maximum response value $\Delta_{p}$, which is selected as the output for the rotation-equivariant descriptor. This operation is referred to as the group alignment operation, where in the trivial representation of dimension C×|G|, all feature values are cyclically connected, allowing features corresponding to different group transformations g to be easily extracted via their indices by shifting the whole descriptor, as illustrated in Figure 4. Finally, an L2 normalization is performed on the descriptor of the first group to produce the final rotation-equivariant dense descriptor output, $D\in R^{C\times H\prime \times W\prime }$.

For the keypoint score at point *p*, after F passes through the *N*×*N* and 1×1 convolutional kernels, the score dimension falls down from C×|G| to |G|, representing the keypoint score responses under different group transformations, g. To obtain a unified and comparable score, a group pooling operation is performed to reduce the dimension to 1, ensuring rotation-equivariant keypoint scores. Finally, all score values are normalized using softmax, resulting in the final dense rotation-equivariant keypoint output $K\in R^{H\prime \times W\prime }$.

### 3.4. Loss Function

During training, the network is provided with two images, $I_{A}$ and $I_{B}$, with a known transformation between them. From these images, the network infers keypoints, descriptors, and orientation histogram information, denoted as $K_{A}$, $D_{A}$, $O_{A}$ for $I_{A}$, and $K_{B}$, $D_{B}$, $O_{B}$ for $I_{B}$, respectively. Since the rotation transformation applied to the original images is known, the group alignment operation can be performed on the original orientational histograms and descriptors. The alignment process is as follows: First, the rotation angle θ of $I_{B}$ relative to $I_{A}$ is determined. Then, the translation required for the group alignment operation (Figure 4) is computed as follows:

(10) $${∆}_{shift}=floor\left(\theta /\frac{360}{\left|G\right|}\right),$$

where |G| is the order of the rotation group. The orientational histogram and descriptor of $I_{B}$ are then aligned using this translation. At this point, the aligned orientational histogram vectors with ground truth data, $O_{A}$, $O_{B}$, and their corresponding descriptors, $D_{A}$, $D_{B}$, are rotation-aligned in the rotation group space, allowing for direct comparison and error calculation.

For any keypoint $p_{A}$ in the overlapping region of $I_{A}$, its descriptor $d_{p}^{A}$ can be extracted, as well as the descriptor $d_{p}^{B}$ of the corresponding keypoint $p_{B}$ in $I_{B}$. The similarity between the two descriptors can be measured using cosine similarity:

(11) $$S(p_{A},\;p_{B})=\frac{d_{p}^{A}\cdot d_{p}^{B}}{\left\|d_{p}^{A}\right\|\left\|d_{p}^{B}\right\|},$$

The probability of matching keypoint $p_{A}$ to keypoint $p_{B}$ is then expressed as follows:

(12) $$P\left(p_{A}\to p_{B}\right)=\frac{exp\left(S\left(p_{A},p_{B}\right)/\tau \right)}{\sum_{q\in K_{B}}exp\left(S\left(p_{A},q\right)/\tau \right)},$$

where $K_{B}$ denotes the total number of keypoints in $I_{B}$, and τ is a temperature parameter that adjusts the sharpness of the probability distribution. Similarly, the probability $P\left(p_{B}\to p_{A}\right)$ (matching a keypoint from $I_{B}$ to $I_{A}$) is defined.

To enforce cycle consistency during matching, which means ensuring that keypoint $p_{A}$ is the best match for $p_{B}$, and $p_{B}$ is the best match for $p_{A}$, the combined matching probability is defined as follows:

(13) $$P_{cycle}\left(p_{A},p_{B}\right)=P\left(p_{A}\to p_{B}\right)\times P\left(p_{B}\to p_{A}\right),$$

which ensures mutual consistency in the matching probabilities between $I_{A}$ and $I_{B}$.

For all keypoint pairs in the overlapping regions of $I_{A}$ and $I_{B}$, if $p_{A}$ and $p_{B}$ are considered a correct match in the ground truth, the directional probability $P_{cycle}\left(p_{A},p_{B}\right)$ should be maximized. The negative log-likelihood (NLL) is used to measure the loss of $P_{cycle}\left(p_{A},p_{B}\right)$:

(14) $$NLL\left(p_{A},p_{B}\right)=-\left(log\left[P\left(p_{A}\to p_{B}\right)\right]+log\left[P\left(p_{B}\to p_{A}\right)\right]\right),$$

By defining all correct match pairs as an index set $\Omega =\{\left(i*,j*\right)\}$, we can calculate the total loss of all descriptors with NLL

(15) $$L_{desc}=\frac{1}{\left|\Omega \right|}\sum_{\left(i*,j*\right)\in \Omega }NLL(i*,j*),$$

here, L_desc represents the descriptor matching loss, which ensures consistent matching probabilities during training by calculating the bidirectional log-matching probabilities.

The similarity matrix S can then be constructed, where S(i, j) represents the similarity between the i-th keypoint of $I_{A}$ and the j-th keypoint of $I_{B}$. If keypoints i and j are a correct match, S(i, j) should satisfy the condition of being the maximum in both its row and column. Hence, an indicator function for keypoints in $I_{A}$ can be defined as follows:

(16) $$y_{i}=\left\{\begin{array}{cc} 1, & \;\;\;\;if\;S\left(i,j\;\right)=\max\left(S\left(i,:\right)\right)\;and\;S\left(i,j\right)=\max\left(S\left(:,j\right)\right)\\ 0, & \;\;\;\;otherwise \end{array}\right..$$

For keypoint detection, let $k_{i}$ represent the predicted “existence probability” for a keypoint in $I_{A}$, derived from the network’s output, $K_{A}$. The detection loss for keypoints can be calculated using binary cross-entropy (BCE):

(17) $$K_{keyA}=BCE\left(y_{A},k_{A}\right)=\sum_{i}-y_{i}log\left(k_{i}\right)-\left(1-y_{i}\right)log\left(1-k_{i}\right),$$

where $y_{i}$ is the ground truth label for whether the i-th point is a keypoint. The total keypoint detection loss is then as follows:

(18) $$L_{key}=\frac{1}{2}\left(L_{keyX}+L_{keyY}\right).$$

For the accuracy of orientation histogram estimation, the alignment loss is calculated using the L2 squared distance between the histogram vectors $o_{P}^{A}$ and $o_{P}^{B}$ for all matched keypoints, $p_{A}$ and $p_{B}$:

(19) $$L_{ori}=\sum_{A,B}{\left|\left|o_{P}^{A}-o_{P}^{B}\right|\right|}^{2}.$$

To ensure sharpness and gradient flow in the orientational histograms, an attention entropy loss is introduced to encourage sharper attention distributions across group dimensions:

(20) $$L_{entropy}=-\frac{1}{2}\left(\sum_{A}o_{P}^{A}\cdot log\left(o_{P}^{A}\right)+\sum_{B}o_{P}^{B}\cdot log\left(o_{P}^{B}\right)\right).$$

Finally, the overall training objective, i.e., the total loss to be minimized, is defined as follows:

(21) $$L_{total}=\lambda_{1}L_{desc}+\lambda_{2}L_{key}+\lambda_{3}L_{ori}+\lambda_{4}L_{entropy},$$

where $\lambda_{1}$, $\lambda_{2}$, $\lambda_{3}$, and $\lambda_{4}$ are hyperparameters that balance the contributions of descriptor accuracy, keypoint detection, and orientational attention precision during training. By weighing and summing up these terms, we achieve balanced supervision across complementary objectives: localization (via $L_{key}$), rotation-equivariance (via $L_{ori}$ and $L_{entropy}$), and descriptor discriminability (via $L_{desc}$).

## 4. Experiment

### 4.1. Implementation Details

As described earlier, the GT-REKD network employs 5×5 and 1×1 equivariant convolutional kernels based on a rotation group with size |G| between the backbone network and detection heads. The choice of the group size |G| follows the conclusions of Han et al. [45], striking an optimal balance between computational efficiency and network performance. Since 3×3 kernels are less effective under 45° equivariant rotations, 5×5 equivariant convolutional kernels were chosen for better performance. Additionally, the bias term in convolutional kernels is removed, and the batch normalization layer is placed before the ReLU activation function to improve training effectiveness. In the backbone encoder network, the output feature channels of each layer in each group are 64, 64, 128, and 128, respectively, which means the final dimension of descriptors is 128. The input to the model consists of cropped grayscale images. During each training batch, the original inputs are randomly rotated counterclockwise by a random angle and augmented with Gaussian noise and other preprocessing operations to form image pairs with known transformations. After extracting outputs from both images in each pair, the error is computed according to Equation (12) and backpropagated to train the network.

### 4.2. Datasets

For training and evaluation, the following three ground texture datasets were selected:

(a) Micro-GPS Dataset:

Released by Zhang et al. [30], Micro-GPS comprises two subsets: PointGrey CM3 (1288 × 964 px, 0.16 mm/pixel, 260 mm above ground): six texture types (fine/coarse asphalt, carpet, concrete, tiles, and wood) covering 145.85 m^2^ (max. single area 41.76 m^2^), with approximately 3900 images per texture; iPhone 6 (1280 × 720 px): two textures (asphalt, carpet), covering 40.27 m^2^ (max. single area 27.52 m^2^).

Both subsets provide image-space poses for global and incremental localization; however, camera intrinsic parameters and ground truth in the world coordinate system are not provided.

(b) HD Ground Dataset:

Published by Schmid et al. [46], HD Ground covers 347.73 m^2^ (maximum single area 106.12 m^2^) across 11 material classes at 1600 × 1200 px resolution (0.10 mm/pixel), containing 128,605 images in total.

The dataset features four main scenes: footpath asphalt, cobblestone parking, office carpet, and kitchen laminate. Also, seven additional surfaces (doormat, bathroom tiles, checker plate steel, garage concrete, ramp rubber, terrace pavement, and workroom linoleum) are included, covering various ground conditions such as dry, wet, and clean surfaces.

The dataset provides homography matrices and 2D Euclidean poses in image coordinates. However, the relatively low sampling frequency results in insufficient overlap between some adjacent frames, which impacts continuous tracking performance.

(c) GeoTracking Dataset:

Published by Xu et al. [35], GeoTracking comprises 17 SLAM-style trajectories (30 Hz) captured with an IDS uEye camera (640 × 480 px, 0.12 mm/pixel) at 0.1 m height, totaling approximately 50,000 frames. Indoor environments (three carpets, granite tile) include 8 trajectories in 4 different texture scenarios, and outdoor environments (brick, coarse asphalt, concrete, fine asphalt, and two gravel roads) include 9 trajectories in 6 different texture scenarios, featuring diverse surface lighting conditions and materials.

Ground truth in world coordinates is provided by a Leica MS60 laser tracker (accuracy ≤ 1 cm), though image frames and timestamps have offsets of ≤50 ms, requiring offline alignment before use.

Table 1 provides a side-by-side comparison of the Micro-GPS, HD Ground, and GeoTracking datasets, summarizing their sensor setups, spatial resolutions, and diversity of sampled scenes. To balance the datasets, 26,000 ground texture images were selected across the three datasets for training and validation, with a 6:4 split between training and validation. During training, images were cropped to 168 × 168 pixels and subjected to rotation group transformations with angles $\theta \in \left[0,2\pi \right)$ to ensure coverage of a wide range of rotation angles. Each input image was artificially augmented by applying random Gaussian noise, motion blur, and varying illumination conditions, thereby enhancing the GT-REKD model’s robustness to these perturbations. Similarly, to ensure the true validity of our algorithm, no data from the training or validation sets was used during the evaluation phase.

### 4.3. Hyperparameter Settings

The training process utilized the ADAM optimizer with a learning rate of 1 × 10^−4^ and the momentum parameters *β*_1_ = 0.9 and *β*_2_ = 0.999. The weight coefficients for the orientation alignment loss (λ_3_) and entropy loss (λ_4_) were set to 15 and 12, respectively, to balance the influence between keypoint loss and descriptor loss. Other weight coefficients were simply set to 1. The output resolution of the dense keypoints, descriptors, and orientational attention maps was 142 × 142, with the top 10,000 keypoints selected for error propagation. Additionally, a temperature coefficient of 0.05 was applied to scale the similarity score matrix S during training.

The training was conducted on two Nvidia RTX 3090 GPUs with a batch size of 4, using mixed-precision training over 50 epochs, with 500 iterations per epoch. To reduce memory usage, the similarity score matrix S was computed in blocks using python library JAX (v0.3.25). The entire training process lasted approximately 7.2 h.

### 4.4. Evaluation Protocol

All quantitative results reported in the experimental results are produced through the following unified evaluation pipeline. The test set defined in Section 3.2 is processed pair-wise. For each image pair, GT-REKD generates dense descriptors, and chooses the topk = 10,000 among them with the highest keypoint scores. To comprehensively reflect the method’s advantages, mainstream handcrafted ground texture feature detectors (ORB, SIFT, SURF, and AKAZE) and representative deep learning-based methods (SuperPoint, DISK, R2D2, and ALIKED) were included as baselines, covering current state-of-the-art techniques. Mutual Nearest Neighbor (MNN) matching was employed for binary descriptors, whereas floating-point descriptors were L2-normalized and then matched by computing dual-softmax probabilities with temperature τ = 0.1 and threshold = 0.7. After that, the surviving correspondence keypoints are denoted as raw matches and provide the input for all subsequent metrics.

During the evaluation of the test dataset, a pair of keypoints/descriptors that truly correspond and are correctly predicted as a match is counted as a true positive (TP); a non-corresponding pair incorrectly predicted as a match is a false positive (FP). Conversely, a corresponding pair missed by the model is labeled a false negative (FN), and a non-corresponding pair correctly rejected is a true negative (TN). With a spatial tolerance of 3 pixels, these four quantities form a confusion matrix, from which we compute the standard metrics.

(22) $$precision=\frac{TP}{TP+FP},$$

(23) $$recall=\frac{TP}{TP+FN},$$

(24) $$F1=2\frac{precision\times recall}{precision+recall}.$$

Precision quantifies the proportion of predicted matches that are truly correct, i.e., low false-match rate; recall measures the proportion of all ground truth correspondences that the model successfully recovers; and the F1-score, the harmonic mean, offers a balanced overall indicator of matching performance.

To evaluate the matching accuracy of different feature detection methods, each correspondence $\left(p_{i},q_{i}\right)$ is treated as correct when the reprojection error is below a pixel threshold *t*,

(25) $$e_{ij}=∥\pi \left(H{\left[p_{i}^{T},1\right]}^{T}\right)-q_{j}{∥}_{2}$$

where $H\left(\cdot \right)$ represents the planar motion between the image pair, given as a homography matrix, and $\pi \left({\left[x,y,z\right]}^{T}\right)={\left[x/z,y/z\right]}^{T}$ represents a projection function. Accuracy is reported as follows:

(26) $$Acc=\frac{\left|\left\{(i,j)|e_{ij}<t\right\}\right|}{\left|\{(i,j)\}\right|}.$$

Repeatability measures a feature detector’s consistency in identifying the same physical point under changes in viewpoint, illumination, or rotation, thereby evaluating its robustness to external variations. Let *P*, *Q* be the keypoint sets detected in the two images. A keypoint $p_{i}\in P$ is counted as repeated if its projection through the ground truth homography H lies within ε=3px of some $q_{i}\in Q$; then, the repeatability is defined as follows:

(27) $$\mathrm{Rep}=\frac{\left|\left\{p_{i}|\exists q_{j},∥{\hat{q}}_{i}-q_{j}{∥}_{2}<\varepsilon \right\}\right|}{min(|P|,|Q|)},$$

where ${\hat{q}}_{i}=\pi \left(H{\left[p_{i}^{T},1\right]}^{T}\right)$.

For each image pair, we can estimate a homography $\hat{H}$ using the raw matches with direct line transform (DLT) and random sample consensus (RANSAC). Since most ground texture setups have negligible scale and shear, we can project $\hat{H}$ onto the closest Euclidean transform $\hat{G}$

(28) $$\hat{G}=\underset{G\in \mathrm{SE}\left(2\right)}{a\mathrm{rg}\min}{\left\|\hat{H}-\lambda G\right\|}_{\;\;\;F},\;\lambda =\frac{1}{2}\left({\hat{H}}_{11}+{\hat{H}}_{22}\right),$$

which yields

(29) $$\hat{\theta }=atan2({\hat{H}}_{21},{\hat{H}}_{11}),{\hat{t}}_{x}={\hat{H}}_{13}/\lambda ,{\hat{t}}_{y}={\hat{H}}_{23}/\lambda .$$

Hence, let $\theta^{*}$, ${t_{x}}^{*}$, ${t_{y}}^{*}$ represent the real pose, then the rotation error can be as follows:

(30) $$e_{\theta }=|\hat{\theta }-\theta^{*}|,$$

and the translation error can be the following:

(31) $$e_{t}=\sqrt{({\hat{t}}_{x}-t_{x}^{*}{)}^{2}+({\hat{t}}_{y}-t_{y}^{*}{)}^{2}}$$

## 5. Results

### 5.1. Rotation Localization Accuracy Analysis

To evaluate the localization capability of the GT-REKD network in various ground texture environments with different rotation angles, this study considers the characteristics of absolute localization tasks for ground textures. Considering the potential differences in rotational matching effects under varying poses, the experimental setup references the format of the Roto-360 dataset [47]. Images not used for training or validation were selected from the three ground texture databases for feature matching to verify the localization performance of GT-REKD. The Roto-360 dataset rotates the same image counterclockwise at 10° increments, generating a total of 36 images with different rotation angles, and provides homography matrices to verify matching performance. Similarly, in this study, ground texture images were rotated by 360°. To avoid interference from black borders after the image block rotated, random rectangular regions were cropped for rotation. This setup tests the effectiveness of different feature extraction methods under various rotational views.

On the ground texture rotation dataset constructed above, GT-REKD achieves average matching accuracies of 96.14%, 96.15%, and 96.16% under 3-pixel, 5-pixel, and 10-pixel error thresholds, respectively—significantly outperforming all baselines. Specifically, it surpasses SIFT (~95.3%) and AKAZE (~94.9%) by approximately 0.8% and 1.2% and improves upon ORB (~66.3%) by nearly 30%. By contrast, deep learning-based methods such as SuperPoint, DISK, R2D2, and ALIKED yield matching accuracies below 30%, representing a performance gap of over 60% relative to GT-REKD. This remarkable robustness stems from the use of group-equivariant convolutional kernels combined with a directional attention mechanism, which endow the network with inherent invariance across the full 0–360° rotation range and enable consistently high-precision matching even under large rotations, repetitive textures, and extremely sparse scenes. Detailed numerical results are presented in Table 2.

**Figure 5:**
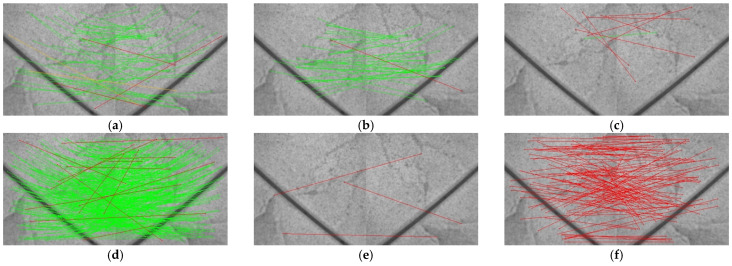

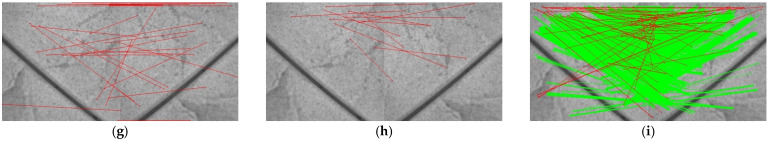
Feature matching performance of GT-REKD and baseline methods under an extremely weak texture scenario; the rotation angle of the image pair is 80°. Green lines represent correct matches (reprojection error < 3px) and red lines represent wrong matches. Subgraphs represent different feature extraction methods: (**a**) SIFT, (**b**) SURF, (**c**) ORB, (**d**) AKAZE, (**e**) SuperPoint, (**f**) ALIKED, (**g**) DISK, (**h**) R2D2, and (**i**) GT-REKD.

Figure 5 presents the matching results of various feature extraction algorithms under an extremely weak texture scenario. Among traditional handcrafted descriptors, SIFT and SURF maintain a moderate number of correspondences with relatively uniform spatial distribution despite large angular displacements, albeit with limited coverage of fine-grained details; ORB—relying on binary descriptors—virtually loses all matching capability under severe rotation; AKAZE extracts a large number of line segments, but these are concentrated in repetitive-texture regions, resulting in high spatial redundancy and increased post-processing overhead. Moving to deep learning-based methods, SuperPoint yields fewer but comparatively accurate matches, whereas DISK and R2D2 increase correspondence counts at the expense of pronounced mismatches (red lines), indicating susceptibility to confusion in highly repetitive or extreme rotation contexts; and ALIKED produces almost exclusively erroneous matches due to inadequate domain adaptation to the ground texture distribution. In stark contrast, GT-REKD inherently encodes full 360° rotation invariance via group-equivariant convolutions, ensuring one-to-one correspondence of planar features irrespective of orientation, and employs a directional attention mechanism to decouple and precisely encode principal orientation information, thereby effectively distinguishing subtle geometric variations within repetitive textures. As shown in the bottom-right panel, GT-REKD achieves a high density of correctly distributed correspondences (green lines) with virtually zero mismatches, providing robust support for subsequent homography estimation or visual odometry.

**Figure 6:**
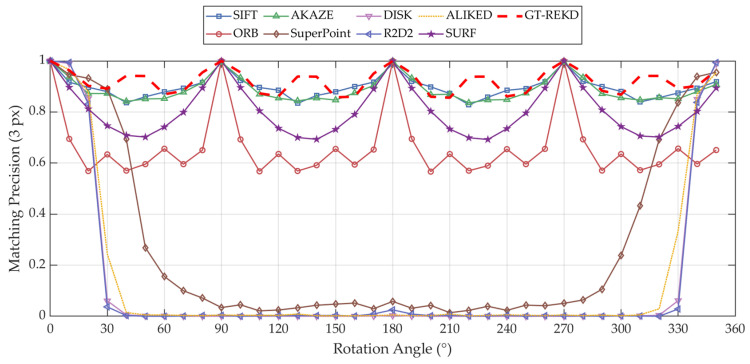
Comparison of matching precision of feature descriptors over full 0–360° rotations.

The curves in Figure 6 reveal that traditional handcrafted methods (SIFT/SURF, AKAZE, and ORB) maintain high matching precision (≥0.9) at principal orientations (0°, 90°, 180°, and 270°) but suffer notable degradation at oblique angles (45°, 135°, 225°, and 315°). Specifically, SIFT/SURF, by leveraging gradient histogram descriptors, incurs only minor fluctuations while preserving precision above 0.8; AKAZE exhibits similar trends with slightly greater amplitude; and ORB, however, experiences precision drops to 0.5–0.6 due to the orientation sensitivity of its BRIEF sampling pattern. In stark contrast, deep learning methods (SuperPoint, DISK, R2D2, and ALIKED) fail rapidly beyond 30° rotation, with precision approaching zero—an outcome of insufficient rotation modeling in both data augmentation and network architecture. By comparison, GT-REKD integrates group-equivariant convolutional kernels with a direction attention mechanism in an end-to-end framework, yielding a near-flat precision curve close to unity across the entire 0–360° range. The equivariant kernels ensure consistent feature responses under all rotations, while the attention module dynamically encodes principal orientation information; joint optimization of keypoint detection and descriptor extraction thus eliminates precision valleys and large-angle failures. Consequently, GT-REKD significantly outperforms both traditional and learning-based baselines in rotational robustness, representing an incredible solution for rotation-invariant feature matching in ground texture scenarios.

### 5.2. Accuracy of Incremental Localization

In this work, incremental localization is implemented using a monocular visual-odometry pipeline: keypoint features detected and matched between successive frames are employed to estimate relative camera poses via planar homography estimation and decomposition with RANSAC-based outlier rejection, producing frame-to-frame trajectories. As introduced in Section 2.1, given matched GT-REKD keypoints between successive frames, we compute the homography H under the planar scene assumption. We then decompose H using the camera intrinsic matrix K and the known camera height *h* above the ground plane to recover the relative rotation R and translation t. This approach ensures a well-posed incremental pose estimate for ground texture imagery.

**Figure 7:**
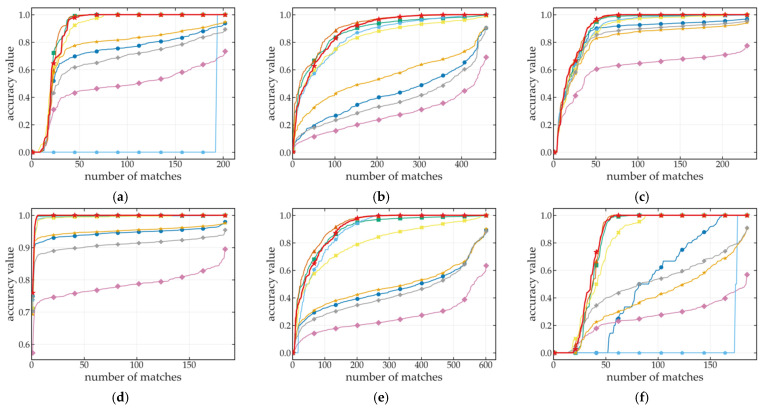

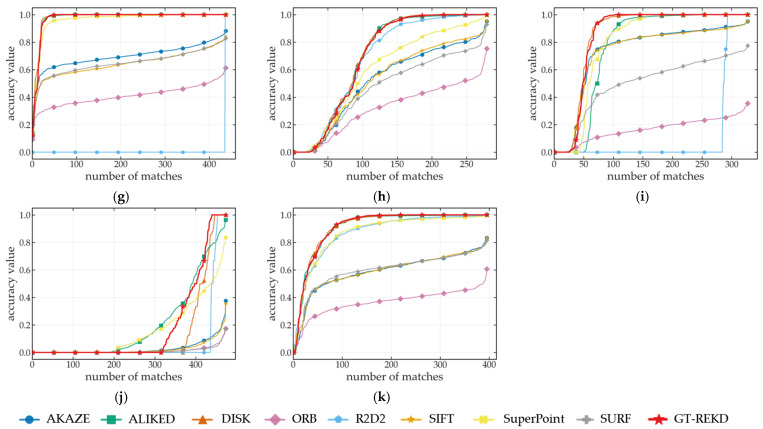
Cumulative distribution of matching accuracy for incremental localization on the HD Ground Dataset. (**a**) Bathroom tiles, (**b**) checker plate steel, (**c**) doormat, (**d**) footpath asphalt, (**e**) garage concrete, (**f**) kitchen laminate, (**g**) office carpet, (**h**) parking place, (**i**) ramp rubber, (**j**) terrace pavement, and (**k**) workroom linoleum.

In the incremental localization scenario, this study analyzed different feature extraction methods with the HD Ground Dataset. We select all indoor and outdoor scenarios to adjust the accuracy of our method, and the cumulative distribution function (CDF) of matching accuracy shown in Figure 7 comprehensively demonstrates the superior performance of GT-REKD in incremental localization scenarios. In richly textured environments—such as doormat carpets, asphalt sidewalks, and concrete garage floors—GT-REKD achieves over 95% accuracy with only 15–25 point correspondences under it, and converges to approximately 99% by 50 correspondences; the resulting CDF exhibits a steep slope and negligible fluctuation, far outperforming traditional methods (e.g., SIFT, SURF), which typically have 60–80 correspondences under similar convergence. Even in the worst texture environment—terrace pavement—the CDF tail of GT-REKD is also shorter than that of other algorithms, indicating that GT-REKD achieves a more concentrated error distribution with fewer extreme deviations, thereby demonstrating superior matching stability and robustness. In contrast, deep learning-based approaches (e.g., SuperPoint, DISK) have more than 150 correspondences below 90% accuracy and display pronounced oscillations in the CDF tail, while R2D2′s performance degrades markedly under high-reflectivity conditions.

**Figure 8:**
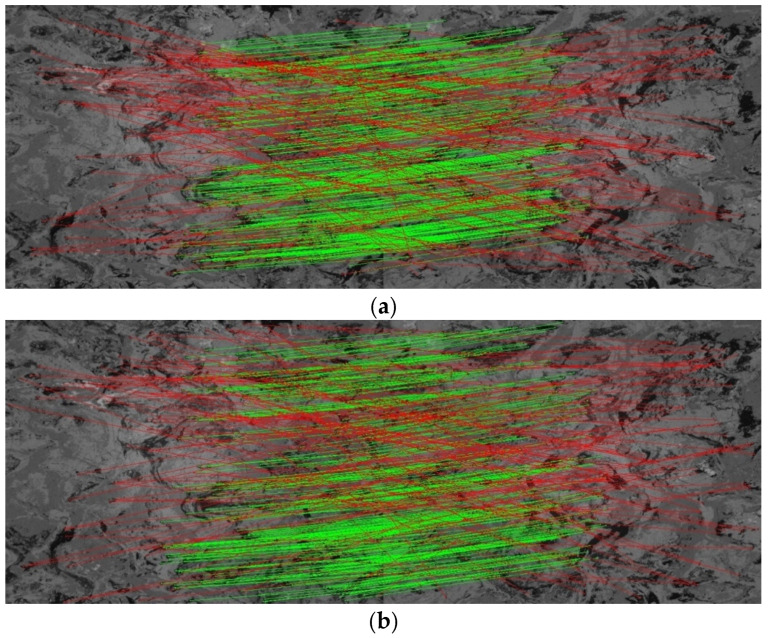

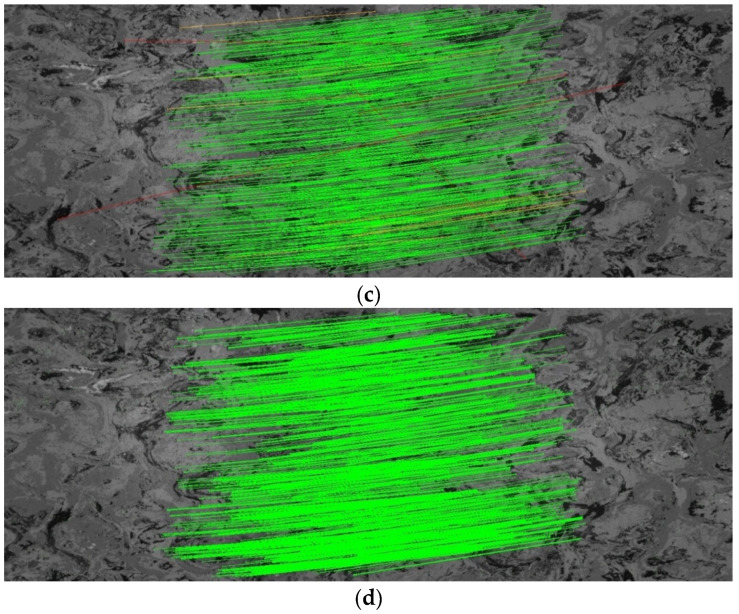
Qualitative feature matching visualization on workroom linoleum surface with different feature extraction methods. Green lines represent correct matches (reprojection error < 3px), and red lines represent wrong matches. (**a**) AKAZE, (**b**) SIFT, (**c**) SuperPoint, and (**d**) GT-REKD.

Figure 8 shows a feature-matching visualization for the workroom linoleum floor scene: green lines denote correctly matched feature pairs produced by each algorithm, while red lines denote incorrect matches. By superimposing these results, the distribution and robustness differences of the various methods on this weakly textured planar surface are revealed. The outcomes are consistent with the main conclusions drawn from Figure 7. Overall, GT-REKD exhibits high accuracy, low cross-scene variability, and compressed long-tail errors, yielding more stable incremental pose estimation.

Following the analysis of multiple ground texture capture sequences from the HD Ground Database, Table 3 presents a quantitative comparison of the overall performance of various feature detection methods. In terms of keypoint detection and matching counts, GT-REKD yields the highest number of detections but exhibits a comparatively low matching rate (≈10%). On the one hand, the matching repetition rate and accuracy have not been reduced; this arises from its use of 1024-dimensional descriptors concatenated over eight rotation-equivariant orientations at the training step for calculating descriptors losses, which reduces discriminability along any single principal orientation and thus lowers individual match scores. On the other hand, this result reflects GT-REKD’s emphasis on detecting a richer set of moderately reliable features. With respect to repeatability, GT-REKD achieves approximately 70%—surpassing traditional methods and matching the performance of deep learning approaches such as DISK—thereby demonstrating robust scene generalization and revisit stability. Finally, across varying error thresholds, GT-REKD maintains high matching precision, particularly under relaxed constraints, yielding a mismatch rate below 6% and significantly outperforming classical techniques. Overall, GT-REKD strikes a notable balance between comprehensive feature detection and localization accuracy, underscoring its suitability for complex and dynamic ground texture localization tasks.

**Figure 9:**
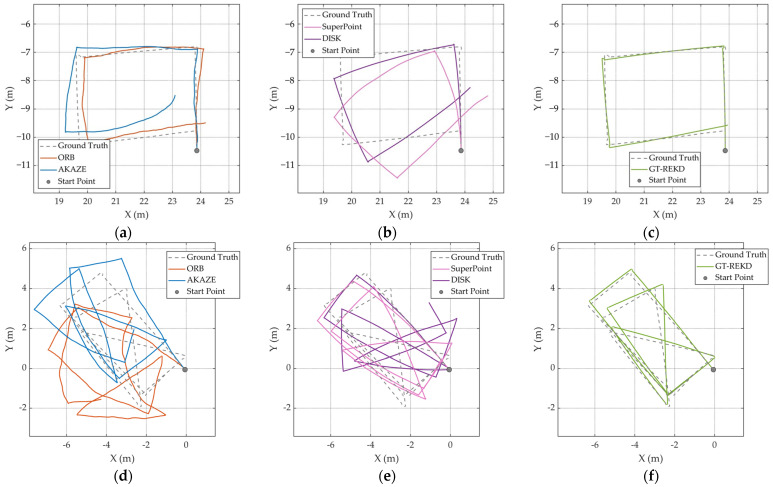
Continuous localization trajectories on coarse asphalt and gravel roads from the GeoTracking Dataset. (**a**–**c**) Refer to coarse_asphalt_seq1; (**d**–**f**) refer to gravel_road2_seq1.

In addition to employing image pairs with known homographies from the HD Ground Database, we further evaluated the continuous localization performance of GT-REKD on real-world trajectories from the GeoTracking Dataset and selected part of famous feature extraction methods for comparison. Figure 9 illustrates two outdoor routes: coarse asphalt, a near-rectangular loop with uniform surface texture, and gravel road, an oblique rectangle characterized by high local texture variability due to loose stones. Conventional handcrafted features (ORB, AKAZE) suffer from feature dropouts in repetitive or sparse-textured regions, resulting in substantial cumulative drift. Deep learning-based features (SuperPoint, DISK) exhibit improved robustness to illumination and scale changes but still accumulate angular deviations over long sequences, preventing precise loop closure. By contrast, GT-REKD trajectories closely coincide with the ground truth, preserving smooth, well-defined corner geometries and achieving low loop closure errors. The integration of rotation-equivariant convolutional kernels and a directional attention mechanism enables stable matching even in low-texture or highly repetitive areas, effectively suppressing drift and maintaining consistently high accuracy across both surface types. These results underscore the feasibility and reliability of GT-REKD for outdoor continuous visual odometry. Table 4 presents a quantitative comparison of the translational alignment errors between the estimated and ground truth trajectories. As the GeoTracking Dataset does not provide exact per-frame ground truth poses, interpolated poses were used for error estimation. The results further corroborate GT-REKD’s adaptability and precision in continuous localization across both indoor and outdoor environments.

### 5.3. Absolute Localization Accuracy Analysis

From the analyses of rotation-based and incremental localization, it is evident that GT-REKD has strong potential for absolute localization scenarios such as relocalization and loop closure detection. To further evaluate GT-REKD’s performance in absolute localization tasks, eleven indoor and outdoor scenes were selected from the HD Ground Dataset. We computed the relative pose transformations between non-adjacent frames using each frame’s prior homography pose matrix and then inferred the image overlap ratios for these non-adjacent pairs. Image pairs with overlap exceeding 30% were chosen from each scene for an absolute localization accuracy analysis, with 100 non-adjacent pairs randomly sampled per scene. In this subsection, we compare the aforementioned feature detection methods in terms of match success rate, rotation-angle error, and translation error.

The results in Table 5 demonstrate that GT-REKD exhibits notable superiority in absolute localization tasks: although its match success rate (57.09%) is lower than that of traditional methods such as SIFT (93.45%) and SURF (92.36%), it substantially outperforms deep learning-based approaches including SuperPoint (22.09%), DISK (15.91%), and R2D2 (5.45%); in terms of rotational error, GT-REKD achieves the lowest mean (5.55°) and a moderate standard deviation (2.53°), indicating exceptional resilience to large rotational variations; similarly, it attains the lowest translational error (4.42 px) with a standard deviation of 2.41 px, confirming its high precision and robustness in non-adjacent-frame pose estimation. Taken together, GT-REKD leads both rotational and translational accuracy, and despite its moderate match success rate, it demonstrates strong potential for high-precision absolute localization and loop closure detection applications that can accommodate partial matching failures.

## 6. Discussion

### 6.1. Ablation Study on Feature Dimensionality and Group Number

To validate the impact of feature dimensionality and group-equivariant sampling on performance, we conducted two ablation experiments under the same training regimen (Section 4.1), varying only the channel width C and the group size |G|. All other hyperparameters remained fixed.

**Figure 10:**
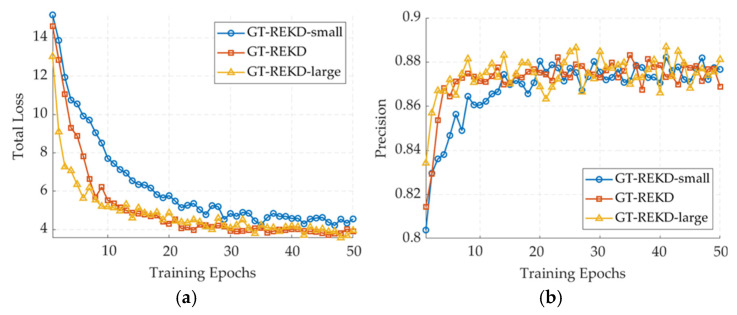

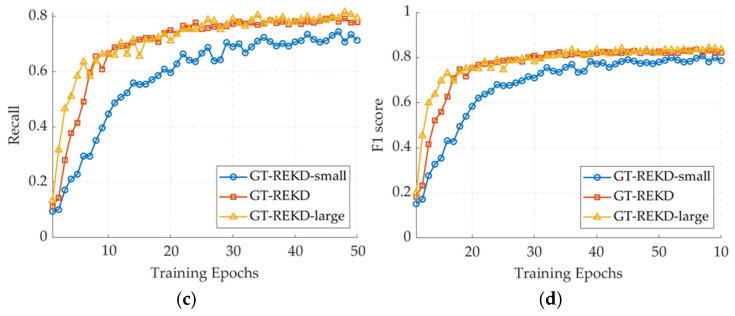
Training dynamics under different channel widths on the validation dataset. (**a**) Total loss; (**b**) precision; (**c**) recall; and (**d**) F1-score.

Building on the preceding description of our training regimen, three variants of GT-REKD were trained with channel widths C = 64, 128, and 256 to validate our feature dimensionality strategy; the first one is referred to as GT-REKD-small, and the last one is referred to as GT-REKD-large, respectively, while all other hyperparameters remain unchanged. As illustrated in Figure 10, with identical training settings, the 128-channel GT-REKD reaches a stable loss of ≈3.8 after roughly 35 epochs, converging faster than the 64-channel variant (GT-REKD-small) and matching the final loss of the 256-channel model (GT-REKD-large). All three configurations surpass 0.86 precision, yet the 128-channel network maintains the highest mean precision with the smallest variance; the 256-channel version attains occasional higher peaks but at the cost of markedly larger variance and double the memory footprint. In recall, the large model leads by <2 percentage points at its peak, a margin the 128-channel model closes by epoch 40 and then holds. Considering the F1-score alongside the inference cost, C = 128 offers the best accuracy–efficiency compromise and is therefore selected for subsequent experiments and real-time deployment.

|G| governs the descriptor’s coverage across 360°. The default |G| = 8 provides equivariance every 45°, yielding the most balanced performance gain. Increasing to |G| = 16 refines the angular resolution but nearly doubles parameters and latency, while complicating convergence of the orientation histogram and raising overfitting risk; reducing to |G| = 4 leaves angular gaps, pushing performance toward hand-crafted schemes such as SURF or AKAZE that rely on a dominant orientation. Hence, |G| = 8 is retained as the optimal choice for balancing performance and efficiency.

### 6.2. Cumulative Drift on Incremental Localization

Figure 9 illustrates the incremental localization trajectories estimated by GT-REKD alongside those of ORB, AKAZE, SuperPoint, and DISK. Unlike the other methods, GT-REKD closely follows the ground truth path throughout both textured and weakly textured segments, exhibiting minimal divergence at loop closures and a smooth, gradual increase in cumulative pose error. This behavior stems from its group-equivariant convolutional descriptors and directional attention module, which together ensure high consistency in keypoint matching across consecutive frames and effectively prevent uncontrolled error accumulation. Also, Table 5 reports GT-REKD’s absolute localization performance, with a mean rotation error of 5.55° and a mean translation error of 4.42 px—significantly better than all baseline methods.

To further suppress drift in a full SLAM system, one may integrate (i) Loop Closure-Driven Pose Graph Optimization, periodically using absolute pose estimates to correct past poses; (ii) Global Bundle Adjustment, performing joint optimization over selected keyframes to redistribute residual error; and (iii) Tightly Coupled Visual–Inertial Fusion (e.g., MSCKF), leveraging IMU constraints to stabilize short-term pose estimates. By combining GT-REKD’s robust frame-to-frame matching with these back-end corrections, a SLAM solution can achieve both high local accuracy and long-term drift mitigation.

### 6.3. Inference Performance and Real-Time Feasibility

After FP16 compilation with TensorRT and execution on a single RTX 3090, GT-REKD achieves an actual average inference speed of approximately 30.63 FPS on 800 × 600 images (i.e., half the native resolution of the HD Ground Dataset). This represents a performance disadvantage relative to conventional methods and some mainstream deep learning approaches, owing chiefly to the high computational complexity of the rotation-equivariant network architecture: additional convolutional and transformation operations are introduced during feature extraction to guarantee rotational invariance while markedly enhancing feature robustness and localization precision. Nonetheless, since existing ground texture localization datasets are typically captured at 60 FPS—with keyframe localization performed every 3–4 frames—GT-REKD, when applied to keyframes in combination with inter-frame tracking, fully meets real-time operational requirements.

## 7. Conclusions

Ground texture localization is an emerging approach that enhances continuity between indoor and outdoor positioning and holds promise for both research advancement and practical deployment. The GT-REKD method proposed in this paper achieves inherent robustness to large-scale rotation underground textures through the collaborative design of group-equivariant convolution and directional attention and demonstrates superior stability and accuracy in both relocalization and incremental localization tasks. By comparing the matching performance of various mainstream feature point methods in different scenarios, the advantages of GT-REKD in terms of feature repeatability, matching accuracy, and rotational invariance are substantiated, making it a strong candidate for visual localization tasks in indoor or outdoor scenarios. In future research, we will further investigate loop detection, IMU fusion, and other related directions to uncover the potential of GT-REKD, and improve the seamlessness, stability, and global consistency of GT-REKD-based ground texture localization.

## Figures and Tables

**Table 1:** Comparative summary of ground texture datasets.

	Micro-GPS	HD Ground	GeoTracking
Image resolution	1288 × 964 px/1280 × 720 px	1600 × 1200 px	640 × 480 px
Pixel size (mm/px)	0.16/0.21	0.10	0.12
Camera height (mm)	260	234	99.8
Number of texture types	4 outdoor/3 indoor	5 outdoor/6 indoor	6 outdoor/4 indoor
Number of frames	26,012	128,605	125,422

**Table 2:** Average matching accuracy (%) under pure rotation conditions for various feature extraction methods. Boldface indicates the highest matching accuracy among all methods.

Feature Extraction Method	3 px	5 px	10 px
SIFT	95.33%	95.38%	95.41%
SURF	91.23%	91.26%	91.27%
ORB	66.29%	66.48%	66.53%
AKAZE	94.92%	95.06%	95.18%
SuperPoint	27.82%	29.11%	29.77%
DISK	13.68%	14.44%	15.35%
R2D2	13.37%	13.50%	13.88%
ALIKED	15.00%	15.31%	15.91%
GT-REKD	**96.14%**	**96.15%**	**96.16%**

**Table 3:** Average incremental localization performance of various feature detection methods. Boldface indicates the highest matching accuracy among all methods.

Methods	Number of Detected Keypoints	Number of Matched Keypoints	Keypoint Redundancy Rate (%)	Matching Accuracy @ 3 px (%)	Matching Accuracy @ 5 px (%)	Matching Accuracy @ 10 px (%)
SIFT	925.14	448.83	54.40%	58.54%	63.03%	64.95%
SURF	989.80	464.06	58.34%	53.51%	57.63%	59.31%
ORB	500.00	216.99	29.89%	35.47%	38.29%	29.40%
AKAZE	758.59	409.09	61.06%	58.56%	62.99%	64.96%
SuperPoint	348.08	137.07	53.32%	77.52%	86.88%	91.91%
DISK	7192.20	1887.16	70.02%	**81.82%**	89.02%	92.34%
R2D2	9981.35	201.78	81.13%	49.63%	56.03%	58.48%
ALIKED	4895.40	2067.06	73.35%	80.96%	88.96%	93.57%
GT-REKD	10,000.98	1025.07	69.92%	81.81%	**89.65%**	**94.08%**

**Table 4:** Translational alignment RMSE (m) on indoor and outdoor GeoTracking Dataset trajectories. Boldface indicates the lowest RMSE among all methods.

	Trajectory	Length (m)	RMSE (m)				
			ORB	AKAZE	SuperPoint	DISK	GT-REKD
Indoor	Carpet1_seq2	38.37	4.966	3.267	3.441	2.617	**2.220**
	Carpet2_seq1	41.47	6.224	2.843	1.929	1.222	**1.168**
	Carpet3_seq1	16.64	5.304	2.280	0.751	0.633	**0.378**
	Carpet3_seq3	45.04	6.581	5.321	7.704	6.212	**1.563**
	Granite_tiles_seq1	27.16	5.020	2.996	2.942	**0.862**	0.937
	Granite_tiles_seq2	40.66	7.559	4.998	2.126	**1.448**	1.473
Outdoor	Brick_seq1	14.06	5.036	1.707	0.728	0.330	**0.204**
	Coarse_asphalt_seq1	15.55	0.471	0.382	1.649	0.797	**0.357**
	Concrete_seq2	23.53	-	2.471	1.133	0.990	**0.536**
	Fine_asphalt_seq1	22.18	1.451	0.724	0.438	0.361	**0.287**
	Gravel_road1_seq1	17.52	1.838	1.113	0.665	0.357	**0.137**
	Gravel_road2_seq1	46.11	1.591	1.020	1.017	0.730	**0.408**

**Table 5:** Absolute localization performance comparison of various feature detection methods. Boldface indicates the lowest relocalization errors among all methods.

Methods	Relocalization Success Rate (%)	Rotation Error (°)		Translation Error (px)	
		Mean	Standard Deviation	Mean	Standard Deviation
SIFT	**93.45%**	7.82	2.60	7.78	2.82
SURF	92.36%	7.07	2.63	7.51	3.24
ORB	88.18%	6.52	2.61	5.11	4.18
AKAZE	86.09%	7.38	2.46	6.88	2.89
SuperPoint	22.09%	9.32	3.18	11.59	3.35
DISK	15.91%	9.12	2.71	11.60	4.02
R2D2	5.45%	6.94	**1.99**	5.68	2.44
ALIKED	22.18%	9.78	2.27	11.84	2.15
GT-REKD	57.09%	**5.55**	2.53	**4.42**	**2.41**

## Data Availability

The original data presented in this study are openly available at https://microgps.cs.princeton.edu/ (accessed on 28 April 2025), https://github.com/JanFabianSchmid/HD_Ground (accessed on 28 April 2025), and https://github.com/sair-lab/GroundSLAM (accessed on 28 April 2025).

## References

[1] Jiang W., Cao Z., Cai B., Li B., Wang J. (2021). Indoor and Outdoor Seamless Positioning Method Using UWB Enhanced Multi-Sensor Tightly-Coupled Integration. IEEE Trans. Veh. Technol..

[2] El-Sheimy N., Li Y. (2021). Indoor Navigation: State of the Art and Future Trends. Satell. Navig..

[3] Mallik M., Panja A.K., Chowdhury C. (2023). Paving the Way with Machine Learning for Seamless Indoor–Outdoor Positioning: A Survey. Inf. Fusion.

[4] Pettorru G., Pilloni V., Martalò M. (2024). Trustworthy Localization in IoT Networks: A Survey of Localization Techniques, Threats, and Mitigation. Sensors.

[5] Li P., Wu W., Zhao Z., Huang G.Q. (2024). Indoor Positioning Systems in Industry 4.0 Applications: Current Status, Opportunities, and Future Trends. Digit. Eng..

[6] Huang J., Junginger S., Liu H., Thurow K. (2023). Indoor Positioning Systems of Mobile Robots: A Review. Robotics.

[7] Zhao C., Song A., Zhu Y., Jiang S., Liao F., Du Y. (2023). Data-Driven Indoor Positioning Correction for Infrastructure-Enabled Autonomous Driving Systems: A Lifelong Framework. IEEE Trans. Intell. Transp. Syst..

[8] Lu Y., Ma H., Smart E., Yu H. (2021). Real-Time Performance-Focused Localization Techniques for Autonomous Vehicle: A Review. IEEE Trans. Intell. Transp. Syst..

[9] Adin I., Mendizabal J., de Miguel G., Goya J., Zamora L., Arrizabalaga S. (2016). Complementary Positioning System in GNSS-Denied Areas. Transp. Res. Procedia.

[10] Iyer K., Dey A., Xu B., Sharma N., Hsu L.-T. (2024). Enhancing Positioning in GNSS-Denied Environments Based on an Extended Kalman Filter Using Past GNSS Measurements and IMU. IEEE Trans. Veh. Technol..

[11] Jarraya I., Al-Batati A., Kadri M.B., Abdelkader M., Ammar A., Boulila W., Koubaa A. (2025). GNSS-Denied Unmanned Aerial Vehicle Navigation: Analyzing Computational Complexity, Sensor Fusion, and Localization Methodologies. Satell. Navig..

[12] Barbieri L., Brambilla M., Trabattoni A., Boulila W., Koubaa A. (2021). UWB Localization in a Smart Factory: Augmentation Methods and Experimental Assessment. IEEE Trans. Instrum. Meas..

[13] Jang B.-J. (2022). Principles and Trends of UWB Positioning Technology. J. Korean Inst. Electromagn. Eng. Sci..

[14] Li W., Chen R., Wu Y., Zhou H. (2023). Indoor Positioning System Using a Single-Chip Millimeter Wave Radar. IEEE Sens. J..

[15] Xu J., Li Z., Zhang K., Yang J., Gao N., Zhang Z., Meng Z. (2023). The Principle, Methods and Recent Progress in RFID Positioning Techniques: A Review. IEEE J. Radio Freq. Identif..

[16] Wang K., Liu Y., Hong Z. (2022). RSS-Based Visible Light Positioning Based on Channel State Information. Opt. Express.

[17] Shang S., Wang L. (2022). Overview of WiFi Fingerprinting-Based Indoor Positioning. IET Commun..

[18] Feng X., Nguyen K.A., Luo Z. (2024). A Review of Open Access WiFi Fingerprinting Datasets for Indoor Positioning. IEEE Access.

[19] Ouyang G., Abed-Meraim K. (2022). A Survey of Magnetic-Field-Based Indoor Localization. Electronics.

[20] Morselli F., Razavi S.M., Win M.Z., Conti A. (2023). Soft Information-Based Localization for 5G Networks and Beyond. IEEE Trans. Wirel. Commun..

[21] Szyc K., Nikodem M., Zdunek M. (2023). Bluetooth Low Energy Indoor Localization for Large Industrial Areas and Limited Infrastructure. Ad Hoc Netw..

[22] Mumuni F., Mumuni A. (2021). Adaptive Kalman Filter for MEMS IMU Data Fusion Using Enhanced Covariance Scaling. Control Theory Technol..

[23] Abaspur Kazerouni I., Fitzgerald L., Dooly G., Toal D. (2022). A Survey of State-of-the-Art on Visual SLAM. Expert Syst. Appl..

[24] Pan C., Li Z., Zhang Q., Soja B., Gao J. (2024). Smartphone-Based Vision/MEMS-IMU/GNSS Tightly Coupled Seamless Positioning Using Factor Graph Optimization. Measurement.

[25] Matsuki H., Murai R., Kelly P.H., Davison A.J. Gaussian Splatting SLAM. Proceedings of the 2024 IEEE/CVF Conference on Computer Vision and Pattern Recognition (CVPR).

[26] Chou C.-C., Chou C.-F. (2021). Efficient and Accurate Tightly-Coupled Visual-Lidar SLAM. IEEE Trans. Intell. Transp. Syst..

[27] Schmid J.F., Simon S.F., Mester R. (2020). Features for Ground Texture Based Localization—A Survey. arXiv.

[28] Kozak K., Alban M. Ranger: A Ground-Facing Camera-Based Localization System for Ground Vehicles. Proceedings of the 2016 IEEE/ION Position, Location and Navigation Symposium (PLANS).

[29] Chen X., Vempati A.S., Beardsley P. StreetMap—Mapping and Localization on Ground Planes Using a Downward-Facing Camera. Proceedings of the 2018 IEEE/RSJ International Conference on Intelligent Robots and Systems (IROS).

[30] Zhang L., Finkelstein A., Rusinkiewicz S. High-Precision Localization Using Ground Texture. Proceedings of the 2019 IEEE International Conference on Robotics and Automation (ICRA).

[31] Wilhelm A., Napp N. Lightweight Ground Texture Localization. Proceedings of the 2024 IEEE International Conference on Robotics and Automation (ICRA).

[32] Wang Q., Pan Z., Hou J., Yu L. (2024). High-Precision Offline Mapping and Localization System Based on Ground Texture with Binary Descriptors. Expert Syst. Appl..

[33] Sheng C., Pan Z., Ye C., Yu L. (2024). Camera Pose Estimation and Relocalization Algorithm Based on Ground Texture. IEEE Trans. Instrum. Meas..

[34] Hart K.M., Englot B., O’Shea R.P., Kelly J.D., Martinez D. Monocular Simultaneous Localization and Mapping Using Ground Textures. Proceedings of the 2023 IEEE International Conference on Robotics and Automation (ICRA).

[35] Xu K., Yang Z., Xie L., Wang C. (2025). GroundSLAM: A Robust Visual SLAM System for Warehouse Robots Using Ground Textures. arXiv.

[36] Schmid J.F., Simon S.F., Mester R. Ground Texture Based Localization: Do We Need to Detect Keypoints?. Proceedings of the 2020 IEEE/RSJ International Conference on Intelligent Robots and Systems (IROS).

[37] Weiler M., Cesa G. (2019). General E(2)-Equivariant Steerable CNNs. arXiv.

[38] Revaud J., Weinzaepfel P., de Souza C.R., Pion N., Csurka G., Cabon Y., Humenberger M. R2D2: Reliable and Repeatable Detector and Descriptor. Proceedings of the 33rd Conference on Neural Information Processing Systems (NeurIPS 2019).

[39] Dusmanu M., Rocco I., Pajdla T., Pollefeys M., Sivic J., Torii A., Sattler T. D2-Net: A Trainable CNN for Joint Description and Detection of Local Features. Proceedings of the 2019 IEEE/CVF Conference on Computer Vision and Pattern Recognition (CVPR).

[40] DeTone D., Malisiewicz T., Rabinovich A. SuperPoint: Self-Supervised Interest Point Detection and Description. Proceedings of the 2018 IEEE/CVF Conference on Computer Vision and Pattern Recognition Workshops (CVPRW).

[41] Gleize P., Wang W., Feiszli M. SiLK: Simple Learned Keypoints. Proceedings of the 2023 IEEE/CVF International Conference on Computer Vision (ICCV).

[42] Lee J., Kim B., Kim S., Cho M. Learning Rotation-Equivariant Features for Visual Correspondence. Proceedings of the 2023 IEEE/CVF Conference on Computer Vision and Pattern Recognition (CVPR).

[43] Karaoglu M.A., Markova V., Navab N., Busam B., Ladikos A. RIDE: Self-Supervised Learning of Rotation-Equivariant Keypoint Detection and Invariant Description for Endoscopy. Proceedings of the 2024 IEEE International Conference on Robotics and Automation (ICRA).

[44] Simonyan K., Zisserman A. (2014). Very Deep Convolutional Networks for Large-Scale Image Recognition. arXiv.

[45] Han J., Ding J., Xue N., Xia G.-S. ReDet: A Rotation-Equivariant Detector for Aerial Object Detection. Proceedings of the 2021 IEEE/CVF Conference on Computer Vision and Pattern Recognition (CVPR).

[46] Schmid J.F., Simon S.F., Radhakrishnan R., Frintrop S., Mester R. HD Ground—A Database for Ground Texture Based Localization. Proceedings of the 2022 IEEE International Conference on Robotics and Automation (ICRA).

[47] Hu W., Tong M. (2023). TRR360D: A Dataset for 360 Degree Rotated Rectangular Box Table Detection. arXiv.

